# Bacteriology and Practice

**Published:** 1889-11

**Authors:** 


### SELECTIONS.

### Bacteriology and Practice.

The relation of microbiology to the advance of knowledge in regard to the prophylaxis and treatment of disease is a matter which is yet by no means clearly defined. Enthusiastic workers in the experimental laboratory are constantly proclaiming the discovery of microscopic parasites in different diseases, and, by the brilliancy of their discoveries, dazzling the minds of the medical profession. But the brilliancy of these discoveries is by no means equaled by improvements in our methods of practice due to them. In fact, there are exceedingly few diseases which are treated better, or more successfully, because of the application of knowledge gained in the bacteriological laboratory. In nothing is this disappointing fact more apparent than in the case of tuberculosis and cholera. The most striking discoveries of many years have been those of the tubercle bacillus and the cholera bacillus; and, what have they done to improve the methods of treatment for or prophylaxis against tuberculosis or cholera? In regard to tuberculosis, the only valuable application of a faith in the etiological relation of the bacillus to the disease is made in surgical practice; and this, valuable as it is, simply confirms principles practically applied long before the bacillus was heard of; while in the treatment of tuberculosis of the viscera nothing whatever has been gained. As before, so now, attention to general hygiene, good air, good food, and roborants are the only means of treating tuberculosis successfully. As to its prophylaxis, the same is true. At present, there is evidence accumulating which indicates that pulmonary phthisis may be infectious, but this is a discovery entirely independent of bacteriological studies, and one which, if true, will be met by measures which have nothing to do directly with the germ theory of disease.

Turning now to cholera, we find about the same state of affairs. Dr. Fernand Roux, of Paris, has recently gone over this subject. He calls attention to the sensation caused by the discovery of the comma bacillus, and the announcement that it is the cause of cholera. What effect, he asks, has this had upon the treatment or prevention of cholera? The imitative genius of Gamaleia has brought forth the suggestion that previous inoculation with attenuated virus of cholera may protect an individual against an attack of the disease. But this suggestion only shows the lack of the logical faculty which seems to possess the disciples of Pasteur. Cholera is not a disease analogous to any in which there is respectable claim for the usefulness of preventive inoculations; and there is no reason—and, of course, no experience—to support the notion that preventive inoculations would have any influence on the development of cholera.

Then, as to treatment: M. Yver suggests corrosive sublimate, administered with the idea of killing the microbes, so as to cure cholera or prevent its development. But the treatment of cholera with mercuric chloride has been tried repeatedly, and gives no results to indicate that it is superior to other methods. Again, as Roux points out, the notion of the prophylactic power of mercury receives a shock from the fact that syphilitics saturated with mercury enjoy no immunity against cholera.

These disappointments in applying the theories of bacteriology to practice in medicine Roux contrasts with the results obtained in the prevention of cholera by methods which leave the particular character of the virus undetermined. These results, he says, are absolutely marvellous, and they have been obtained by the English physicians in India by observation alone and the study of epidemics, without a single theory being advanced. From 1826 to 1844 there died, on an average, annually from cholera 35 per 1,000 inhabitants. At the present time the mortality has fallen to 2 per 1,000. This almost incredible result has been obtained by substituting filtered water for that which was employed without taking into consideration from whence it came. Everywhere where this substitution was effected the cholera has diminished in such proportions that it may be admitted that the Europeans in India are protected against the disease if they do not commit any imprudence. At Calcutta, before 1870, the average mortality from cholera was 10.1 per 1,000. Since this period, from which dates the establishment of pipes of filtered water, the mortality is 3 per 1,000, having diminished by more than two-thirds. This result can only be attributed to the distribution of filtered water to the inhabitants. That which absolutely proves this fact is that in December, 1877, one of the principal reservoirs was seriously damaged, in consequence of which the distribution of filtered water was suspended for some time. The cholera, which before the accident was at its normal rate, assumed an ascending progression, and diminished as soon as filtered water was redistributed for consumption. It is true that enthusiastic microbiologists may say that it is precisely the microbe discovered by Koch, which propagates the cholera, and that consequently the prophylaxy of the malady proceeds from this fact. It may, however, be observed that a great number of English physicians, and those not among the least eminent, refuse to accept the ideas of Koch. The filtered water distributed at Calcutta does not pass through filters capable of retaining a microbe as small as the comma bacillus. It may, therefore, be said that the prophylaxy of cholera was known, and put in practice, for some time in all parts of India by the English before the comma bacillus was thought of.

The great value of bacteriological work lies chiefly in the accumulation of observations which may some day be put to practical use, and which already give shape to our ideas of pathology, and in surgery have lead to the establishment of methods of cleanliness which have revolutionized the results of many operations. Bacteriological work is of great scientific interest, and has in it the promise of greater usefulness than it has yet attained; but nothing is gained by misunderstanding or misrepresenting its attainments up to the present time.

---

Dr. H. M. Whelpley says that those who make many balsam mounts at a time soon find that a number of clips are required to hold the cover glasses in position until the balsam hardens. The clips in the market cost from seventy-five cents per dozen upwards. He has found it much cheaper and just as convenient to make his own clips from ordinary hair-pins, as proposed by Professor Wall. Such clips will cost about five cents per dozen.

